# An End-User Audit of Reproducibility, Data Leakage, and Overfitting of the Top-Ranked ADMET Prediction Models in TDC Leaderboards

**DOI:** 10.1021/acs.jcim.6c00819

**Published:** 2026-07-02

**Authors:** Ihor Koleiev, Roman Stratiichuk, Nazar Shevchuk, Mykola Melnychenko, Alex Nyporko, Daniil Todoryshyn, Vladyslav Husak, Sergii Starosyla, Semen Yesylevskyy, Alan Nafiiev

**Affiliations:** † Receptor.AI Inc., 20-22 Wenlock Road, London N1 7GU, U.K.; ‡ Department of Physics of Biological Systems, Institute of Physics of the National Academy of Sciences of Ukraine, 46 Nauky Ave., Kyiv 03038, Ukraine; § Department of Biophysics and Medical Informatics, Educational and Scientific Centre “Institute of Biology and Medicine”, Taras Shevchenko Kyiv National University, 64 Volodymyrska Str., Kyiv 01601, Ukraine; ∥ Department of Cellular, Computational and Integrative Biology, The University of Trento, Via Sommarive 9, Povo, Trento 38123, Italy; ⊥ Institute of Organic Chemistry and Biochemistry, Czech Academy of Sciences, Prague 6 CZ-166 10, Czech Republic; # Department of Physical Chemistry, Faculty of Science, Palacký University Olomouc, 17. Listopadu 12, Olomouc 771 46, Czech Republic; ∇ Taras Shevchenko Kyiv National University, 64 Volodymyrska Str., Kyiv 01601, Ukraine

## Abstract

Public leaderboards such as the Therapeutics Data Commons (TDC) ADMET benchmark are widely treated as a ranking of state-of-the-art models. However, a high leaderboard position is only meaningful if the corresponding model can actually be reproduced and deployed by an independent researcher. In this work, we audit whether the top-ranked TDC ADMET models meet that bar. We assessed the top-ranked models of all 22 TDC ADMET leaderboards from the perspective of an end user with access only to the publicly released artifacts of each model—its publication, code repository, and installation instructions. For every end point, the top three models were screened with a unified protocol including an execution environment reproducibility check, a data-leakage assessment, verification of the hyperparameter-optimization procedure, and a reevaluation against the current leaderboard. Only three models (CaliciBoost, MapLight, and MapLight + GNN) passed all stages and reproduced their reported performance. The remaining models failed because of unavailable code, nonreproducible environments, runtime incompatibilities, or methodological flaws. We traced direct or indirect data leakage in the MiniMol, GradientBoost, and XGBoost models, and used deliberately overfitted variants of our own Mol2Vec-based models to show that tuning on the public test set—whether accidental or intentional—can substantially inflate both metrics and leaderboard rank. These results indicate that current TDC leaderboard positions cannot be read as a direct measure of model quality and practical applicability and emphasize the urgent need for better public ADMET benchmarks based on the hidden test sets, strict data set versioning and model submission with standardized inference environments.

## Graphical abstract

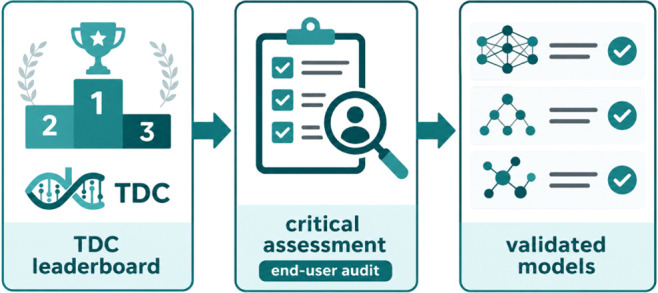

## 1 Introduction

The process of novel drug development is lengthy, costly, and associated with a high risk of failure at late stages.^1,2^ One of the primary reasons for attrition (accounting for up to 50% of failures) is not insufficient therapeutic efficacy, but rather unfavorable pharmacokinetic and toxicological properties, collectively referred to as ADMET (absorption, distribution, metabolism, excretion, and toxicity).^3–5^ Understanding the ADMET profile at early stages of drug development is critically important for rational drug design, as it enables the elimination of inherently nonviable compounds before performing expensive *in vivo* testing.^6^ Preliminary evaluation of chemical libraries allows researchers to focus on regions of chemical space that exhibit an optimal balance between biological activity and safety,^7,8^ thereby dramatically reducing both drug development time and costs.

Historically, several computational approaches have been developed for ADMET assessment, ranging from classical “drug-likeness” rules^9^ to quantitative structure−property relationship (QSAR) methods based on linear regression. However, with the accumulation of large-scale data in public databases (such as ChEMBL, PubChem, and ZINC) and the increasing complexity of prediction tasks, the focus has shifted toward machine learning (ML) methods. Today, ML models are capturing an ever-growing segment of the drug discovery landscape, gradually displacing traditional physicochemical simulations.^10^ This trend is driven by their ability to capture nonlinear relationships in high-dimensional data and their high prediction throughput, which is suitable for large-scale screenings.^11–13^ The most recent ML ADMET prediction models exploit novel neural network architectures such as graph neural networks (GNNs)^14,15^ and transformers.^11^

With rapid proliferation of ADMET prediction algorithms, an urgent need has emerged for rigorous and fair comparison of their quality.^16,17^ Traditional evaluation strategies include cross-validation and testing on external data sets using standard performance metrics (RMSE, MAE for regression; AUC-ROC, F1-score for classification).^18^ While these approaches are implemented in widely used libraries such as scikit-learn and DeepChem, their application to chemical data requires careful consideration of the features of chemical space structure: models often demonstrate excellent performance on familiar molecular scaffolds but lose predictive power when confronted with fundamentally novel chemotypes.^19–21^

Dedicated online ADMET benchmarking platforms have gained significant traction in recent years. Notable examples include MoleculeNet,^22^ PharmaBench,^23^ and arguably the most well-known Therapeutic Data Commons (TDC).^24^ The TDC platform is a comprehensive resource that provides access to curated ADMET data sets with predefined data splits and introduces a public leaderboard for ADMET prediction models. As an open data platform, TDC is often referred to as the “gold standard” for the academic community, allowing researchers to instantly compare their results with the cutting-edge rivals.^25,26^

However, the availability of fully open data sets is both an advantage and a serious drawback. Open train-test split introduces the risk of deliberate cheating or unintentional model adaptation to a specific test set.^27,28^ When a test set remains publicly available for years, researchers may implicitly optimize architectures or hyperparameters to maximize performance on that specific data set, without guaranteeing robustness under real-world conditions involving truly novel data.^29^ Moreover, some recent ML models reach prediction accuracy for certain end points that exceeds the accuracy of the experimental assays used to generate the training data. This phenomenon is a clear indicator of overfitting: the model captures data set-specific noise or systematic measurement errors rather than genuine biological or chemical relationships.^30,31^

Researchers may resort to intentional overfitting because they need to demonstrate state-of-the-art results to get their articles accepted by high-ranking journals, obtain research grants, boost their own careers or promote commercialization of the models in the drug discovery market. Regardless of the reason, inflated leaderboard positions diminish scientific value of the benchmarks and hamper the progress in the field. To flag overfitting, it is important to understand what the maximum “fair” accuracy of predicting ADMET properties is. Given experimental uncertainty and interlaboratory variability,^32^ the coefficient of determination R^2^ for most biological end points rarely exceeds 0.7−0.8.^33^ As emphasized by domain experts, any model claiming accuracy beyond this threshold on noisy biological data is likely learning random fluctuations in a particular data set rather than fundamental chemical principles.^34,35^

Thus, at the current stage of cheminformatics development, there is an urgent need for a critical assessment of existing ADMET benchmarking practices. The field must transition from merely recording leaderboard rankings toward the development of robust evaluation methodologies that explicitly account for data uncertainty^16,36^ and prevent deliberate or unintentional data manipulations.

The objective of the present study is to assess the TDC ADMET leaderboards from the standpoint of an end user—an academic or industrial researcher who attempts to apply a top-ranked leaderboard model using only the artifacts its authors have released publicly. Rather than an expert-level reanalysis of each model’s internal design, we ask a deliberately practical question: for the models that occupy the top of each leaderboard, can their reported performance be reproduced in practice, and does it survive scrutiny for data leakage and test-set overfitting? We therefore examined the top three entries of every TDC ADMET end point along three axes: the reproducibility of model training and inference procedures from the public artifacts, the robustness of the training protocol against train−test data leakage, and the presence of overfitting or other inflation of the reported metrics. We restrict the analysis to the top three models per end point as the most obvious choices for an end user. We show that several top-ranked TDC models have serious problems in one or more of the above-mentioned categories and therefore cannot be regarded as state-of-the-art despite their outstanding reported metrics. As an example of a properly developed and fairly evaluated model, we also report our in-house Mol2Vec-based models, which achieve competitive leaderboard positions without any of these flaws.

## 2 Materials and Methods

Among the ADMET property prediction models displayed on the TDC leaderboards, top models were identified for each of the 22 ADMET end points presented here. After comparing the lists of top models and identifying/removing duplicates, a final list of models was formed for subsequent quality assessment. The models were checked according to a procedure including verifying model reproducibility, assessing potential data leakage, and checking the correctness of model optimization. At each stage, models that did not meet the relevant criteria (see below) were eliminated, saving resources and evaluation time. Only models that successfully passed all stages of preliminary testing were admitted to evaluation using the complete set of ADMET end points. The ML model we developed for evaluating ADMET properties was also tested on this same complete set.

### 2.1 Analysis of ADMET Benchmarks

#### 2.1.1 Selection of Leaderboard Models

For each ADMET end point, we focused on the top three leaderboard models because benchmark analyses typically concentrate on the small group of leading methods rather than the full ranking. In many TDC end points, performance differences among the top models are small, so restricting the analysis to the top three captures the main competitive approaches while avoiding over-representation of closely related variants. This choice therefore allows the study to remain both representative of state-of-the-art performance and practically feasible for detailed verification. After removing duplicates across end points, this procedure resulted in a final set of ten representative architectures: ADMETrix, CFA, GradientBoost+, MapLight, Maplight + GNN, MiniMol, CaliciBoost, SimGCN, XGBoost, and ZairaChem.

This strategy ensured that the analysis focused on models that are competitive according to the community of TCD benchmarks and, simultaneously, allows rational use of resources and time for obtaining maximum useful information. For consistency with the end-user perspective, we did not extend the inclusion criteria beyond the top-3 in response to models eliminated at earlier validation stages because, in our framing, an unreproducible top-ranked model is itself a finding worth reporting.

#### 2.1.2 ADMET End Points

To evaluate the models, all 22 end points available within the TDC ADMET benchmark leaderboard were involved. These end points span the full ADMET spectrum—absorption, distribution, metabolism, excretion, and toxicity—and include both regression and classification tasks with task-specific evaluation metrics (MAE, Spearman correlation, AUROC, AUPRC). A complete list of the end points, data set sizes, and metrics is provided in Supplementary Material (Table S1). For individual models, evaluation was stopped early if significant issues (e.g., impossibility to code running, suggested data leakage or unclear procedure of hyperparameter tuning) were encountered at earlier validation stages.

#### 2.1.3 Data Set Sources and Splits

All data sets and splits were obtained directly from the TDC ADMET Benchmark Group via the canonical tdc.benchmark_group.admet_group loader. The Benchmark Group exposes a single, fixed train/test partition per end point − a scaffold split with 20% of compounds held out for the test set − and is the partition against which all entries of the TDC ADMET leaderboard are evaluated. We emphasize that, although the broader TDC data set API (tdc.single_pred.ADME) allows a user to choose between several split strategies (random, scaffold, cold-start, combination), the Benchmark Group used for leaderboard submissions does not expose this choice: only the scaffold split with the fixed test set is accepted. We therefore used this single, leaderboard-tied split throughout the study, with no modification, for all 22 ADMET end points. The standard loader call is
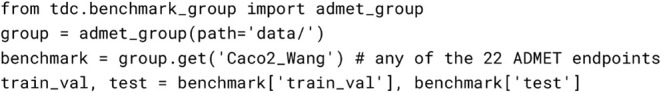


The same loader is used unchanged in the code released alongside this manuscript (see Data and Software Availability).

We deliberately did not apply any in-house structure standardization, deduplication, or filtering to the official TDC splits, since any such modification would alter the evaluation set and break the direct comparability between our reproduced metrics and the published TDC leaderboard values that constitute the reference point of this study. The orthogonal question—whether the TDC splits themselves contain residual duplicates or noisy labels that may inflate metrics for all models—has been addressed independently in the literature and is summarized in the Discussion. An exception was the MiniMol model, which is a foundation model pretrained on the LargeMix data set. In this case, the LargeMix pretraining set was filtered according to the developers’ recommendations by removing all molecules present in the TDC test sets, thereby preventing direct information leakage from pretraining to evaluation.

### 2.2 Evaluation Protocol for Leaderboard Models

All selected leaderboard models were assessed using a unified, multistage protocol designed to ensure reproducibility, methodological correctness, and fair comparison. Throughout the evaluation we adopt the perspective of an end user − an academic or industrial researcher who attempts to apply a leaderboard model in their own ADMET pipeline using only the materials made publicly available by its authors (paper, code repository, installation and usage instructions, declared dependencies). Accordingly, we did not try to resolve installation or runtime issues proactively by contacting the original authors of the models, opening GitHub issues, requesting undocumented patches, or obtaining nonpublic versions of the code. The ability of the public artifacts alone to support reproducible deployment is treated as part of what is being evaluated, on the grounds that practical usability is an inherent component of “state-of-the-art” claims. This choice deliberately favors a stricter and more pragmatic view of reproducibility over a more lenient one based on author-mediated assistance.

### 2.3 Reproducibility Check

Models were tested in a local environment. Reproducibility was assessed using a single end point per model: specifically, the end point on which the model achieved its highest leaderboard rank. For this end point, the reported leaderboard metric was recomputed and compared with the published value. Given the sensitivity of some models to random seeds and software versions, minor numerical deviations in absolute metric values were considered acceptable, provided that they did not affect the model’s relative ranking on the leaderboard; only systematic discrepancies leading to rank changes were considered as essential.

### 2.4 Assessment of Data Leakage

To assess potential data leakage between training and test data sets, Tanimoto similarity scores were computed using Morgan fingerprints (radius 3, 2048 bits). Pairwise similarities were calculated between all molecules in the training and test sets. Two complementary statistical metrics were analyzed: median similarity, reflecting the overall overlap between chemical spaces, and maximum similarity, highlighting the presence of highly similar or identical molecules (Tanimoto ∼ 1). For the MiniMol model, similarity analysis was conducted between the pretraining data set and the union of all TDC test molecules. An additional qualitative check was performed by querying PubChem for known ADMET-related proteins associated with the compounds from the pretraining data set.

### 2.5 Verification of Optimization Procedures

Primary publications and software repositories were examined to verify that hyperparameter optimization procedures were explicitly described and that no tuning or model selection was performed on the test sets. This step is essential because all TDC test sets are publicly accessible, making intentional or accidental overfitting to a test set possible.

### 2.6 Final Evaluation

Models that passed the previous stages were evaluated following the official TDC ADMET Benchmark Group instructions (https://tdcommons.ai/benchmark/admet_group/overview/), using the fixed scaffold-based train/test split described in Section 2.1.3. The resulting metrics were compared against all leaderboard entries available at the time of writing (November 2025), and model rankings were recomputed accordingly.

We did not apply formal statistical significance tests (e.g., Friedman/Nemenyi-style posthoc tests on per-fold metrics, as recommended by Ash et al.^32^) to the reproduced leaderboard. Such tests are most informative when applied to a fairly populated and unbiased set of methods, whereas the set of models surviving our end-user reproducibility protocol is small and explicitly selection-biased toward models with usable public artifacts. A complementary, statistically grounded comparison of TDC ADMET leaderboard methods has been already independently reported by Kamuntavičius et al.,^37^ to which we refer the interested reader.

### 2.7 Development of In-House ML Models

To complement the analysis of leaderboard models, the in-house ML models were developed and evaluated using the TDC protocol.

### 2.8 Model Architecture

LightGBM (Light Gradient Boosting Machine) was selected as the base learning algorithm due to its computational efficiency, efficient handling of sparse molecular fingerprints, and consistently strong baseline performance in QSAR and ADMET prediction tasks. LightGBM implements gradient boosting decision trees with histogram-based split finding, which enables efficient training even for high-dimensional and heterogeneous molecular feature spaces. Regression tasks were handled using LGBMRegressor^38^ API, while classification tasks employed LGBMClassifier^39^ API with a binary cross-entropy objective.

### 2.9 Molecular Representations

Mol2Vec embeddings were used as a fixed baseline representation across all benchmarks. Mol2Vec is an unsupervised embedding method inspired by distributional semantics models in natural language processing. In this approach, molecular substructures are treated analogously to words, while molecules are represented as sentences of such substructures. Embeddings are learned such that substructures occurring in similar chemical contexts are mapped to nearby points in a continuous vector space.

Mol2Vec embeddings were generated using a Skip-gram Word2Vec model trained on a large and chemically diverse set of 883,897,271 small molecules. Substructures were identified using Morgan fingerprints with radius 0 and 1, and the resulting vocabulary encompassed 13,278 unique “words.” Each molecule was represented by aggregating the embeddings of its constituent substructures into a single 512-dimensional vector. Substructures not present in the training vocabulary were assigned a default embedding corresponding to the mean vector of the embedding space.

In addition to Mol2Vec, diverse sets of molecular fingerprints (23 types, Table S2) were used as complementary feature groups to define the optimal feature set for calculation of each end point. In addition, benchmark-specific sets of features “filtered_descs” were generated by combining RDKit 2D and Mordred 2D descriptors with subsequent filtering. The filtering procedure included removing duplicate descriptors, excluding descriptors with calculation errors, low-variance filtering, and rejecting features with high correlation (cross-correlation filtering). Descriptor filtering was performed using training data only to avoid information leakage.

### 2.10 Optimization Pipeline

Model optimization followed a two-phase procedure. In Phase 1, Sequential Forward Selection (SFS) was applied to identify an optimal set of molecular feature groups for each benchmark. Starting from a fixed Mol2Vec baseline, additional feature groups were iteratively added based on 5-fold cross-validation performance. The selection criterion was the selection_score value, calculated as the difference between the mean value of the appropriate metric and the corresponding standard deviation.

In Phase 2, Bayesian hyperparameter optimization was performed using the Optuna framework^40^ with a Tree-structured Parzen Estimator sampler.^41^ Hyperparameters were optimized using the same cross-validation protocol as in Phase 1. Optimization was skipped for benchmarks where the model already achieved top leaderboard rank after feature selection alone. Performance on a test set was monitored exclusively for verification purposes and was never used for feature selection or hyperparameter tuning.

### 2.11 Final Training and Evaluation

For each benchmark, the final model was retrained on the full training set using the selected features and optimized hyperparameters, and then evaluated on the TDC test set. To explicitly quantify the contribution of successive optimization stages, all 22 ADMET end points were evaluated three times for the in-house models: (i) using the baseline (nonoptimized) model, (ii) after the feature selection stage (Phase 1 optimization), and (iii) after hyperparameter optimization (Phase 2 optimization).

### 2.12 Development of Deliberately Overfitted Variants of In-House ML Models

To evaluate the possible overfit influence on the performance metrics and appropriate model ranks in TDC ADMET leaderboards, deliberately overfitted variants of In-house ML models were constructed. Overfitting was introduced by using the TDC test data sets instead of TDC train data sets during the both SFS and HPO optimization phases. Resulting overfitted models have features sets and hyperparameters different from honest ones (see Supplementary Tables S3 and S4).

## 3 Results

### 3.1 Results Summary

Table 1 summarizes the architecture and additional training data of each candidate model. Across the top-3 entries we screened, the leaderboard is dominated by fingerprint+descriptor combinations fed into gradient-boosted decision trees (MapLight, MapLight + GNN, CaliciBoost, GradientBoost+, XGBoost, ADMETrix), with a smaller contingent of GNN-based or hybrid systems (MiniMol, SimGCN, ZairaChem) and one ranking-fusion meta-method (CFA). The top-1 position on the majority of end points is currently held by methods built around classical molecular representations rather than by end-to-end graph neural networks or large self-supervised foundation models − a pattern consistent with recent independent observations that fixed, hand-crafted representations remain competitive with, and often outperform, learned ones on TDC ADMET tasks at the current data scale.^37^ The two exceptions in our top-3 set − MiniMol (a 10M-parameter foundation model pretrained on the LargeMix corpus) and SimGCN (a graph similarity GCN) − were the models that we found prone to problems in the reproducibility analysis below, and in MiniMol’s case to direct pretraining-set leakage into the TDC test sets (Section “Assessment of data leakage”). Table 2 summarizes the outcomes of our four-stage validation protocol for all ten candidate models, with the specific reason for elimination at each stage. Only three models − CaliciBoost, MapLight, and MapLight + GNN − passed all four stages and entered the final reevaluation.

Two architecture-level observations follow from Tables 1 and 2. First, the three models that passed every stage of our protocol are all built around fixed, hand-crafted molecular representations (descriptors and classical fingerprints) fed into gradient-boosted decision trees. Even MapLight + GNN, which incorporates a learned GIN component, uses it as a feature extractor rather than as an end-to-end predictor, with the actual prediction still performed by CatBoost. Second, models built on more recent representation-learning paradigms − a foundation model (MiniMol), a similarity GCN (SimGCN), and a multiencoder ensemble that includes a chemical language model (ZairaChem) − all failed at various stages of reproducibility pipeline. We do not interpret this as evidence that learned representations are inherently weaker than classical ones on ADMET tasks, rather, it is consistent with the observation that classical-representation pipelines tend to be simpler to release reproducibly and harder to overfit to a particular benchmark, while the more complex learned-representation systems are simultaneously more dependent on careful curation of their pretraining data, more sensitive to execution environment, and more attractive targets for inadvertent test-set tuning. The complementary statistical comparison performed by Kamuntavičius et al.,^37^ which found classical-representation models to be at least competitive with their learned-representation counterparts on TDC ADMET, points in the same direction.

**Table 1:** Architecture and Training Data of the Top-3 Leaderboard Models Considered in This Study^a^

**Model**	**Family**	**Molecular representation**	**Predictor**	**Additional training data beyond TDC train split**	**TDC end points covered**
**CaliciBoost**	Classical ML/AutoML	PaDEL, Mordred, RDKit (2D + 3D) descriptors (selected via systematic feature comparison)	AutoML pipeline (FLAML); winning configuration is gradient-boosted trees on PaDEL/Mordred 2D + 3D descriptors.	OCHEM Caco-2 data set (used for separate cross-evaluation, not for TDC submission)	1 (Caco-2 only)
**MapLight**	Classical ML	ECFP counts (1024) + Avalon counts (1024) + ErG (315) + 200 RDKit physicochemical descriptors (≈2,573 dim total)	CatBoost (gradient-boosted decision trees)	None—fixed feature set across all 22 end points	22
**MapLight + GNN**	Classical ML + pretrained GNN	MapLight features + 300-dim GIN supervised-masking embeddings from a pretrained graph isomorphism network (≈2,873 dim)	CatBoost (gradient-boosted decision trees)	GIN encoder pretrained on a public DGL-LifeSci corpus; no extra TDC-task data	22
**MiniMol**	Foundation model (pretrained GNN)	Learned graph embeddings	Fine-tuned on each TDC end point	LargeMix pretraining corpus: PCBA-1328, L1000-MCF7, L1000-VCAP, PCQM4M-G25, PCQM4M-N4 (∼6 M molecules)	22
**SimGCN**	GCN	Similarity-based graph convolutional network	End-to-end	Not specified in public artifacts	Multiple
**ZairaChem**	Hybrid/AutoML ensemble	Multiple molecular encoders incl. ChemGPT (language-model embeddings) and classical fingerprints	Stacked ensemble (incl. TabPFN component)	Encoder-specific pretraining sets	Multiple
**ADMETrix**	Classical ML (hybrid)	Mixed fingerprints + descriptors	Gradient-boosted ensemble	None declared	Multiple
**CFA (Combinatorial Fusion Analysis)**	Meta-method (rank fusion)	Operates on the ranked outputs of other models	Score-and-rank fusion	Inherits from the underlying base models	Multiple
**GradientBoost+/XGBoost**	Classical ML	Fingerprints + descriptors (author-defined)	GBDT/XGBoost	None declared	Multiple

a Sources for architecture details: Notwell and Wood^42^ for MapLight and MapLight + GNN; Le et al.^43^ for CaliciBoost; the GitHub repositories cited in Materials and Methods Section 2.1.1 for the remaining models. “TDC end points covered” refers to the number of leaderboards on which the model’s authors submitted entries.

**Table 2:**
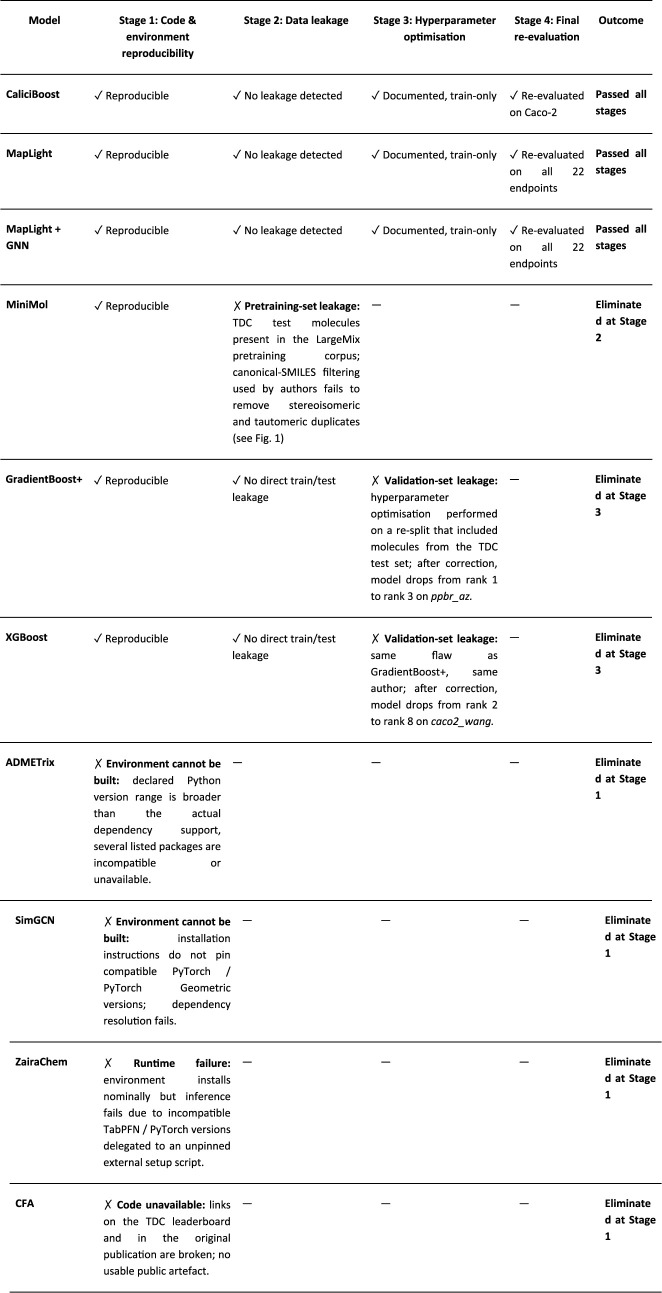
Outcome of the Four-Stage Validation Protocol for Each Candidate Model^a^

a “—” indicates that the corresponding stage was not entered because the model had already been eliminated at an earlier stage.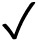 = passed; 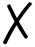 = failed.

### 3.2 Checking Availability and Reproducibility of the Models

First, we assessed the code availability and possibility to reproduce the results and have found critical issues in a number of models. Source code of the model **CFA** (Combinatorial Fusion Analysis) model^44^ was not accessible due to the broken links on both the TDC Web site and the corresponding paper. This model ranks first in the *clearance_hepatocyte_az* and *half_life_obach* leaderboards, second in the *bbb_martins* and *herg* leaderboards, and third in the *dili* and *vdss_lombardo* leaderboards.

For **ADMETrix** ^45^ (first in the *ld50_zhu*, second in the *ames* and *lipophilicity_astrazeneca*, and third in the *cyp2c9_veith*) and **SimGCN** models^46^ (third in the *herg*) the execution environments could not be reproduced based on the available installation instructions. In the case of ADMETRix, the provided setup description specifies a broad Python version requirements, whereas the dependency set implicitly assumes a narrower and internally inconsistent range of versions; as a result, several required packages were either incompatible or unavailable, preventing successful environment construction. For SimGCN, the installation instructions do not specify a compatible version-matched combination of PyTorch and PyTorch Geometric extensions. This leads to unresolved dependencies and makes environment construction impossible. Fragments of the logs with the corresponding errors are shown in the Supplementary Information.

**ZairaChem** model^47^ (first in the *ames*, second in the *cyp2c9_substrate_carbonmangels* and *dili*, third in the *bioavailability_ma*) was successfully deployed, but execution failed due to incompatibilities among core libraries in the inference pipeline. In particular, the installation procedure delegates environment setup to an external script without fixing compatible versions of PyTorch and the TabPFN components used by the model, which results in runtime import errors despite nominally successful installation. The relevant log fragment containing a description of the errors encountered is provided in the Supplementary Information.

These findings emphasize the lack of proper software development practices, testing and quality control, which is, unfortunately, common in the academic machine learning community and hinders adoption and cross-validation of developed models.

### 3.3 Assessment of Data Leakage between Training and Test Data Sets

**MiniMol** is one of the most successful models, which is ranked first on seven TDC leaderboard (*bbb_martins, bioavailability_ma, cyp2c9_substrate_carbonmangels, dili, hia_hou, lipophilicity_astrazeneca and solubility_aqsoldb*). MiniMol is a foundational model pretrained on the massive Graphium **LargeMix** data set containing approximately six million unique molecules. According to the authors, molecules present in all TDC ADMET test sets were removed from the pretraining corpus to prevent data leakage. However, the paper does not specify precisely how this matching and filtering were performed, which raises concerns about potential data leakage

The LargeMix data set comprises three types of data: transcriptomic data constituting about 5% of the pretraining set (L1000 VCAP and L1000 MCF7), biochemical assay data constituting about 41% (PCBA-1328), and quantum chemical data constituting about **54%** (PCQM4M G25 and PCQM4M N4 data sets).

Following the description in the original paper, canonical SMILES were used to remove molecules belonging to the TDC test sets from the MiniMol pretraining corpus. This results in exclusion rates consistent with those reported by the MiniMol developers—7% for MCF7, 4% for VCAP, and 0.6% for PCBA. However, inspection of similarity distributions (Figure 1A−C) demonstrates the presence of molecules that are highly similar or identical to ones in the TDC test set across all three analyzed MiniMol training data sets with the largest number of such molecules observed in the PCBA data set.

Upon closer inspection it became evident that simple filtering by canonical SMILES is insufficient, as it often fails to account for molecular chirality properly. It removes only one specific stereoisomer from the data set, while alternative stereoisomers and/or representations of the same compound with missing stereochemistry annotations are retained and lead to a data leakage. For example, danazol is present as a concrete stereoisomer in the TDC all-test data set, yet it was not removed from the MiniMol pretraining data set during filtering because its stereochemistry was not explicitly specified in the MiniMol training set (Figure 1d). Tautomerism may also contribute to the persistence of undetected duplicates, as different tautomeric forms of the same compound are usually represented by distinct canonical SMILES strings. Consequently, molecules that are chemically equivalent but differ only in tautomeric state may bypass filtering and contribute to unintended data leakage. This is consistent with the broader curation literature, where canonical-SMILES matching has been shown to systematically miss duplicates that a proper structure-standardization pipeline would expose.^37,48^

**Figure 1:**
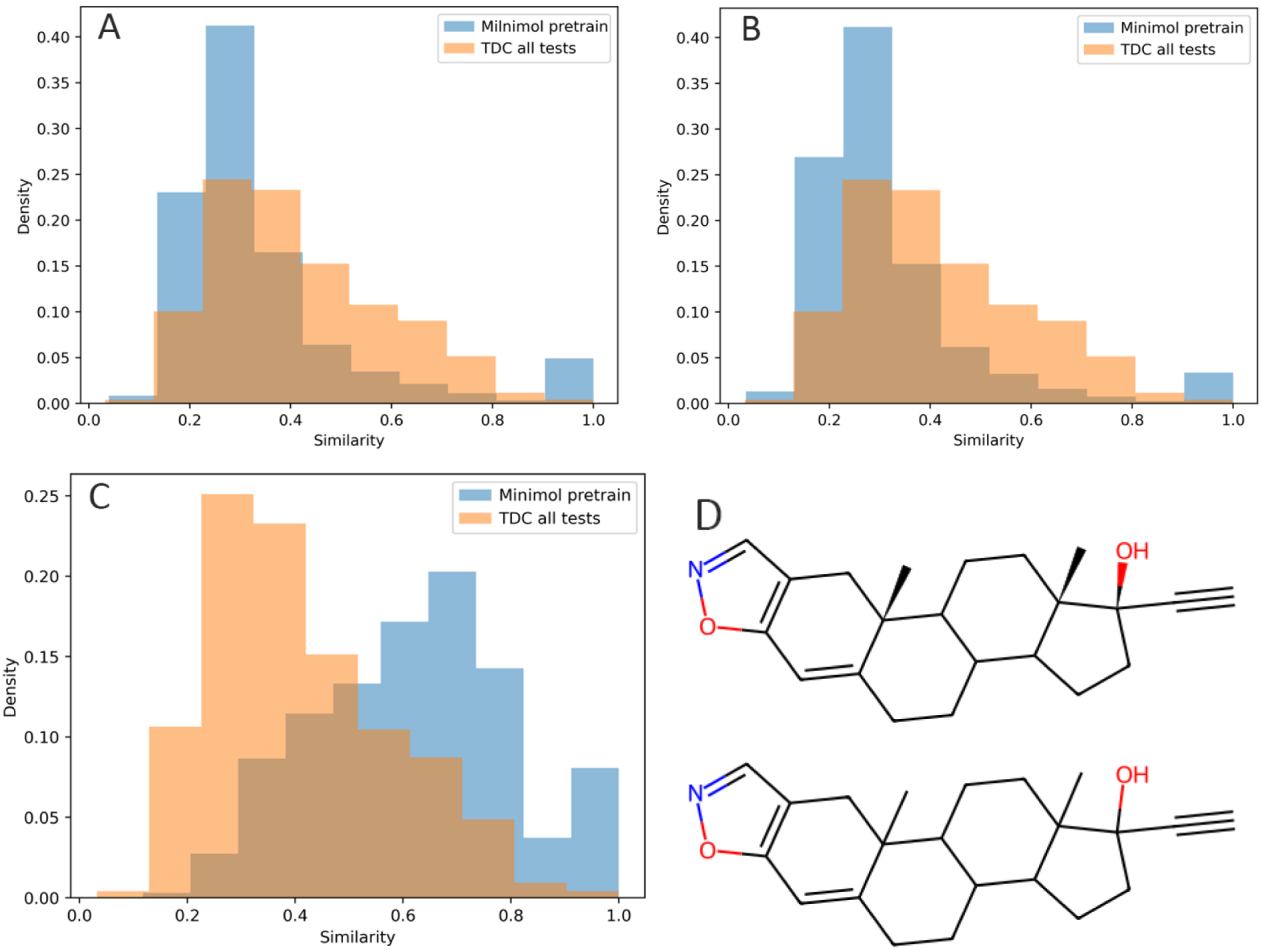
Distribution of similarities between MiniMol pretrain and TDC test sets. For each TDC test molecule, the histogram shows the maximum Tanimoto similarity (Morgan fingerprint, radius 3, 2048 bits) to any molecule in the comparison set. (A) MCF7, (B) VCAP, (C) PCBA. (D)—danazol molecule as found in TDC all-test data set (with chirality annotations, top) and MiniMol pretrain data set (without chirality annotations, bottom).

### 3.4 Verification of Hyperparameter Optimization Procedures

At this validation stage, significant issues were detected in two models—**GradientBoost** and **XGBoost**, both developed by the same author. GradientBoost ranks first on the *ppbr_az* leaderboard, whereas XGBoost ranks second on the *caco2_wang* leaderboard. For both models we identified a flaw in the data set splitting procedure applied during hyperparameter optimization: the validation sets generated at the model tuning stage partially overlapped with the (public) TDC test sets. This issue originates from an incorrect function call that, prior to hyperparameter optimization, resplits the full data set randomly into training, validation, and test subsets with the default hard-coded random seed of 42. As a consequence, the validation set contains, among others, molecules from the original TDC test set constituting a direct data leakage. The similarity distributions between the test set and the validation sets used by the model authors and corrected by us are shown in Figure 2.

Although the core model training procedures are implemented correctly in both cases, the data leakage at the hyperparameters optimization leads to inflated reported leaderboard metrics. After correcting the split function and reevaluating the models we obtained substantially lower leaderboard ranks—third place for GradientBoost (decrease from first) and eighth place for XGBoost (decrease from second).

**Figure 2:**
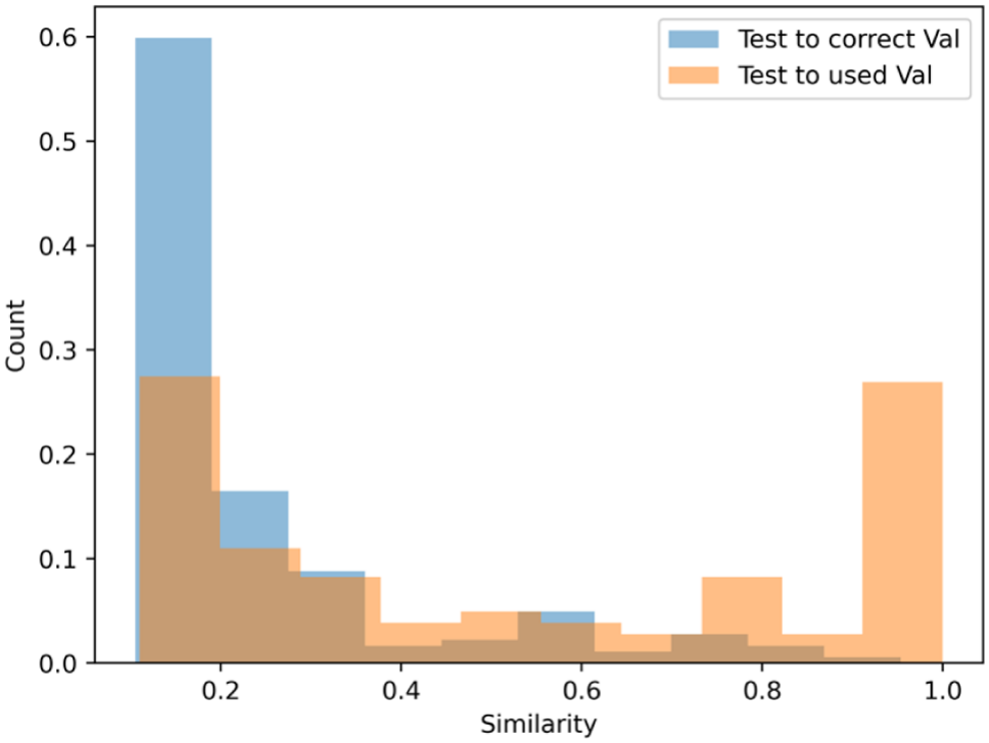
Distribution of similarities between TDC caco2 test set and the validation set used during tuning vs a correctly generated validation set. For each TDC test molecule, the histogram shows the maximum Tanimoto similarity (Morgan fingerprint, radius 3, 2048 bits) to any molecule in the respective validation set.

### 3.5 Re-Evaluation of Model Performance

Only three models reached the final testing stage: **CaliciBoost** (first on the *caco2_wang*), **MapLight + GNN** (first on *clearance_microsome_az, cyp2c9_veith, cyp2d6_veith, cyp3a4_veith, herg, pgp_broccatelli,* and *vdss_lombardo*), and **MapLight**. CaliciBoost was developed exclusively for the evaluation of Caco-2 effective permeability. Our MAE estimate for CaliciBoost was 0.271 ± 0.002, compared with 0.256 ± 0.006 reported in TDC, while the model retained its first-place ranking. The small discrepancy between these values is most likely related to the updates of the TDC data sets, which are, unfortunately, not under version control. That is why it is not possible to attribute the benchmarking results to a particular well-defined data set version unambiguously. Part of observed discrepancies may also be related to differences in computational hardware and/or software environments used for evaluation. MapLight and MapLight + GNN were evaluated across all 22 TDC ADMET end points. A summary of their reevaluated performance is shown in Table 3.

**Table 3:**
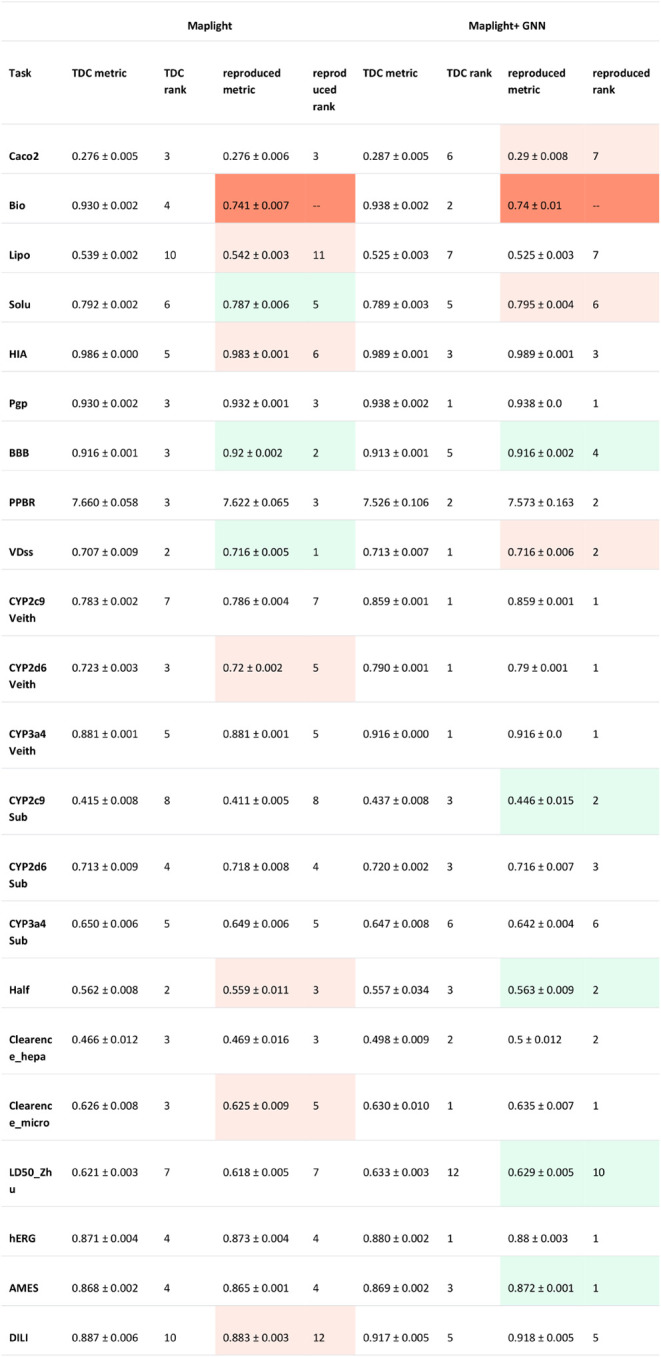
Original and Re-Evaluated Performance Metrics and Ranks of Maplight and Maplight + GNN Models on Different TDC ADMET End Points^a^

a Reproduced rank got better 
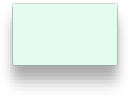
. Reproduced rank got worse 
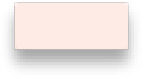
. Reproduced rank got much worse
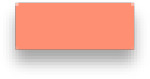
.

MapLight + GNN retains its leaderboard rank in 13 out of 22 ADMET end points, improves its position in five cases (reaching first place for *ames*), and exhibits a lower rank in three cases. A substantial decline in performance is observed only for *bioavailability_ma*. Similarly, MapLight preserves its original rank in 11 end points, improves its ranking in four cases (including attaining first place for *vdss_lombardo*), and shows a lower rank in six cases. As with MapLight + GNN, a pronounced drop in performance is again observed for *bioavailability_ma*.

Across all 22 end points, the absolute differences between TDC-reported and reproduced metrics are very small (<0.01) for the vast majority of cases for both models, indicating high overall reproducibility of the leaderboard results. For most ADMET tasks, reproduced metrics fall within overlapping uncertainty ranges of the TDC values, and rank changes (where present) are small and consistent with the narrow performance gaps among top-ranked models. Minor deviations are likely caused by routine technical factors such as random seed variability, numerical precision, or differences in software and hardware environments used for evaluation. It should also be noted that the TDC platform does not provide explicit data set or leaderboard versioning. As a result, minor changes in data set composition, preprocessing, or split definitions over time may contribute to small discrepancies between reproduced and leaderboard metrics, even when identical evaluation protocols are followed.

In general, MapLight + GNN tends to outperform MapLight, particularly for metabolism-related end points (*cyp2c9_veith, cyp2d6_veith, cyp3a4_veith,* and *clearance_microsome_az*), where MapLight + GNN consistently occupies top leaderboard positions and maintains close agreement between reproduced and TDC metrics. For several end points—including *hERG, pgp_broccatelli, and cyp3a4_veith*—the reproduced ranks for both models closely match the TDC leaderboard positions, highlighting stable and reproducible performance in these tasks.

The largest and systematic discrepancies between TDC and reproduced results are consistently observed for *bioavailability_ma*, where both models exhibit markedly lower reproduced AUROC values and substantial rank changes. A second, but less pronounced, deviation is observed for *ppbr_az*, where MAE differences are larger than for other end points, although rankings remain comparatively stable. Given that TDC does not maintain publicly versioned data set releases, it is possible that the benchmark snapshot used to obtain the original leaderboard results differs from the data set currently available. Such untracked updates (e.g., removal/addition of molecules or adjustments to train-test splits) could contribute to systematic shifts in performance for particular end points.

### 3.6 Performance Testing of In-House Models

The summarized results of our in-house model performance evaluation across 22 ADMET end points are presented in Table S3. For the majority of end points, the two-stage optimization procedure—Sequential Forward Selection (SFS) followed by Bayesian hyperparameter optimization (HPO)—leads to improved predictive performance compared with the baseline models, as reflected in better evaluation metrics and/or higher leaderboard rankings. This indicates that, in most cases, systematic feature selection combined with targeted hyperparameter tuning is an effective strategy for enhancing model performance within the LightGBM framework. Notable exceptions are *dili a*nd *pgp_broccatelli*, for which the baseline variants of the models remain the most competitive; and *bbb_martins, cyp3a4_substrate_carbonmangels, clearance_hepatocyte_az, clearance_microsome_az,* and *ames,* for which the models after the first optimization stage (SFS) achieve the best performance among the three tested variants. In the case of *bbb_martins*, SFS substantially improves the model position from eighth to second place on the leaderboard, whereas subsequent HPO results in a marked drop in ranking despite comparable or slightly improved metrics.

For *ppbr_az* and *half_life_obach*, performance temporarily deteriorates after SFS but improves after HPO, ultimately yielding better metrics and rankings than those of the corresponding baseline models. This suggests that, for these end points, feature selection alone is insufficient, whereas hyperparameter tuning is critical for achieving optimal performance. In the case of *hia_hou*, the optimized model reaches first place on the corresponding leaderboard. In addition, the optimized models for *ld50_zhu, lipophilicity_astrazeneca, solubility_aqsoldb*, and *cyp2c9_veith* rank within the top five positions of their respective leaderboards, indicating consistently strong performance across both physicochemical and metabolism-related tasks.

Several binary classification end points feature very high reported AUROC values on the TDC leaderboards. In *hia_hou, bbb_martins,* and *pgp_broccatelli*, a substantial fraction of leaderboard models exhibit AUROC values above 0.9 (17 out of 25 models for *hia_hou*, 12 out of 25 for *bbb_martins*, and 9 out of 25 for *pgp_broccatelli*). The AUROC values obtained for our optimized models on these end points also fall within this high-performance range. This suggests that achieving AUROC values above 0.9 on these benchmarks should *not* automatically be interpreted as evidence of overfitting or test-set leakage and can, at least in part, be a normal performance regime for these tasks. As Walters^49^ discusses, recurring high apparent performance on such data sets is often more parsimoniously explained by data set-level factors (narrow chemical-space coverage, label-conflicting duplicates, assay-artifact-driven signal, and heterogeneous end point definitions) than by model-side leakage. This remains an open question for the affected TDC end points and is, in our view, a natural target for future data set-focused work. None of this rules out the presence of overfitted models in the TDC ADMET leaderboards, which is the model-side concern we address in the rest of this paper.

### 3.7 The Effect of Deliberate Model Overfitting

In order to study the effect of possible unintentional or deliberate model overfitting to the test set we constructed deliberately overfitted versions of our in-house models and analyzed their ranking patterns in comparison with those of other top-performing models such as MapLight + GNN and MiniMol. Table S4 summarizes the performance of deliberately overfitted in-house models across all 22 TDC ADMET end points. In contrast to the “honest” models, the overfitted variants were intentionally tuned to maximize performance on the public TDC test sets, allowing us to examine how sensitive leaderboard rankings are to extreme, test-set−driven optimization. For each end point, we consider the best achieved leaderboard position across the three variants (baseline, SFS, and HPO).

For a substantial fraction of benchmarks, deliberate overfitting leads to marked improvements in leaderboard positions, often elevating models from the middle or lower tiers directly into the top ranks. The most pronounced effects are observed for *bbb_martins* (rank 8 → 1), *clearance_microsome_az* (14 → 1), *cyp2d6_substrate* (12 → 1), and *ames* (13 → 1). Similarly strong gains are seen for *caco2_wang* (9 → 2), *cyp3a4_substrate* (14 → 2), *dili* (13 → 2), and *hia_hou* (2 → 1). For another group of end points, overfitting improves performance more moderately, consistently moving models upward in the leaderboard without necessarily reaching first place. This behavior is observed for *lipophilicity_astrazeneca* (13 → 5), *solubility_aqsoldb* (7 → 3), *ld50_zhu* (6 → 3), *half_life_obach* (11 → 9), *and ppbr_az* (9 → 5, then 7 after HPO).

In contrast, bioavailability_ma remains largely insensitive to deliberate overfitting: all three variants (baseline, SFS, and HPO) retain rank 21 despite noticeable changes in AUROC values. This likely indicates that our model architecture is generally unsuitable for this end point. On the other hand, the issue may originate from the benchmark itself. Specifically, it is possible that the data set was updated without public disclosure, resulting in the inability to reproduce the leaderboard metrics reported for the top-ranked models before (see our verification results for MapLight + GNN and MapLight, both of which failed to reproduce the reported performance on this end point).

Quantitatively, deliberate overfitting produces a substantial upward shift in leaderboard positions: the overfitted model reaches first place in 5/22 end points, second place in 3/22, and third place in 2/22, i.e., it appears in the top three in 10/22 end points overall (≈45%) (Table 4). In contrast, our conventionally trained (“honest”) in-house models reach the top three only twice across the same 22 end points (best rank across baseline/SFS/HPO), indicating a pronounced gap between standard optimization and test-set−driven tuning.

**Table 4:** Best Metrics and Ranks of In-House Models across Different TDC ADMET End Points

**benchmark**	**metric name**	**honest metric**	**honest rank**	**overfitted metric**	**overfitted rank**
caco2_wang	MAE	0.2875	7	0.2694	2
bioavailability_ma	AUROC	0.6744	21	0.7147	21
lipophilicity_astrazeneca	MAE	0.5108	5	0.5112	5
solubility_aqsoldb	MAE	0.7808	5	0.7735	3
hia_hou	AUROC	0.9938	1	0.9951	1
pgp_broccatelli	AUROC	0.902	10	0.9202	7
bbb_martins	AUROC	0.9225	2	0.9376	1
ppbr_az	MAE	7.9457	7	7.779	7
vdss_lombardo	Spearman	0.4949	11	0.4909	13
cyp2c9_veith	AUPRC	0.7922	5	0.792	5
cyp2d6_veith	AUPRC	0.7178	7	0.7085	5
cyp3a4_veith	AUPRC	0.8748	9	0.8826	5
cyp2c9_substrate_carbonmangels	AUPRC	0.3459	19	0.3566	18
cyp2d6_substrate_carbonmangels	AUPRC	0.6842	10	0.7566	1
cyp3a4_substrate_carbonmangels	AUROC	0.6384	9	0.6658	2
half_life_obach	Spearman	0.3927	9	0.4089	9
clearance_hepatocyte_az	Spearman	0.3353	16	0.4413	6
clearance_microsome_az	Spearman	0.5542	14	0.6314	1
ld50_zhu	MAE	0.5866	4	0.5766	3
herg	AUROC	0.8032	11	0.8495	6
ames	AUROC	0.8422	10	0.8723	1
dili	AUROC	0.86	16	0.9422	2

Considering the TDC ADMET leaderboards at the time of the study, the top-three ranks are dominated by a small set of recurring models rather than a large, diverse pool of methods. Among these, MapLight appears in the top three 17 times (1 first place, 5 s, and 11 thirds), MapLight + GNN 15 times (7 firsts, 5 s, and 3 thirds), and MiniMol 13 times (7 firsts, 4 s, and 2 thirds), with additional recurrent contributions from CFA (8 appearances), ZairaChem,^5^ and ADMETrix.^4^

Our deliberately overfitted model reaches the top three in 10 out of 22 end points, demonstrating that test-set−driven tuning can elevate leaderboard positions but can not dominate all leaderboards. By comparison, MapLight (17/22), MapLight + GNN (15/22), and MiniMol (13/22) appear in the top three substantially more often than any of our in-house overfitted models tested here. This suggests that the recurrent TDC leaders are likely to be superior in the choice of model architecture, descriptor set or training pipeline, rather than by deliberate fitting to a specific end point.

## 4 Discussion

The present study reveals systematic limitations in using the open TDC leaderboard as a direct indicator of ADMET model quality. Our reevaluation of top-ranked TDC models shows that full reproducibility is achievable for only three methods—CaliciBoost, MapLight and MapLight + GNN, while all other top-ranked models are prone to serious technical or methodological issues. At the same time, our experiments with deliberately overfitted in-house models demonstrate that accidental or deliberate model tuning to the public test set can substantially alter leaderboard positions, often elevating otherwise mediocre models to top ranks.

Some models that occupied the TDC ADMET top-3 at the time of writing could not be reliably used in practice due to unavailable source code, impossibility to set up an execution environment, inability to run the model in a properly configured environment or critical flaws in data processing and optimization procedures. This points to a structural weakness in the current leaderboard format: high ranks are not accompanied by systematic verification of code availability, functionality, and methodological correctness.

An analysis of the model repositories showed that reproducibility issues in many cases stem from mistakes and omissions in the installation and running instructions. This signifies insufficient scrutiny in software development and distribution practices, which is, unfortunately, common in academic software development in general and in the ML field in particular. The availability of a public repository alone does not guarantee the practical reproducibility of models in the absence of complete, well-defined and well-tested installation instructions.

The reproducibility issues reported here characterize the public artifacts of the models (such as code, instructions, and declared dependencies) rather than an intrinsic limitation of the underlying methods. In many cases, direct correspondence with the original authors would likely have allowed us to resolve installation and runtime problems and to evaluate the models further. We deliberately did not take this route, since our goal was to assess what a typical end user, with limited technical knowledge and the tight time constraints, can achieve with the materials publicly associated with each leaderboard entry. From this perspective, the inability to deploy a top-ranked model from its public artifacts is itself a substantive finding, and we believe it complements, rather than competes with, evaluations conducted under expert-mediated conditions.

Our analysis shows that although only about 30% of the selected models reached the final stage of verification, this subset overall demonstrates good reproducibility. For MapLight, MapLight + GNN, and CaliciBoost, discrepancies between reported and reproduced metrics were small and likely caused by technical factors (differences in hardware, software environments, or library versions). This confirms that, when implemented correctly, top TDC models can be reproducible, but simultaneously highlights that a substantial fraction of leaders does not meet this standard.

A complementary, methodologically distinct line of work has explicitly addressed the question of whether differences between TDC leaderboard methods are statistically significant. Kamuntavičius et al.^37^ integrated cross-validation with a posthoc multiple-comparison test based on the studentized range distribution and reported that many of the small performance gaps separating top-ranked TDC ADMET entries are not statistically distinguishable. The methodology recommended by Ash et al.^32^ provides a more general, well-justified framework for such pairwise method comparisons. Together, these works suggest that, even setting aside the reproducibility and leakage issues we report here, leaderboard ranking alone (in isolation from significance testing) should not be treated as a definitive ordering of methods. We did not repeat such an analysis on our own reproducible subset, since its small size and obvious selection bias toward models with usable public artifacts would make significance estimates difficult to interpret.

Comparison of our in house “honest” models with the ranges of TDC metrics shows that their values consistently fall within the characteristic ranges of the corresponding benchmarks. This indicates that the metric ranges reported on the leaderboards are broadly realistic and are not merely a consequence of pervasive overfitting.

However, a markedly different picture emerges when analyzing the sensitivity of leaderboards to overfitting. Our deliberately overfitted models entered the top-3 in 10 out of 22 end points, whereas “honest” models did so in only 2 out of 22. This contrast demonstrates that public leaderboards are highly susceptible to accidental or deliberate overfitting to the open testing data set.

At the same time, leading TDC models (MapLight − 17/22, MapLight + GNN − 15/22, MiniMol − 13/22 top-3 appearances) appear in the top ranks far more frequently than any of our own overfitted variants. Such dominance in a broad range of end points is difficult to reproduce within a single model architecture even under deliberate overfitting. Such dominance across a wide range of end points cannot be easily explained by tuning for specific end points, as models such as MapLight use a fixed set of descriptors that is the same for all tasks. Rather, this consistency suggests that the selected descriptors represent molecular properties that influence multiple ADMET processes, allowing the same modeling pipeline to perform well across different end points. The model authors do not disclose how exactly this particular set of descriptors was selected, but in terms of model performance, it appears to be quite successful.

These conclusions align with reports indicating that long-lived open test sets encourage gradual adaptation of models to a specific benchmark, even without deliberate “cheating”.^50,51^ Moreover, in some cases the reported accuracy may exceed the reproducibility of the underlying experimental measurements themselves, which can serve as an indicator of hidden overfitting.^30,31^

One of the crucial findings of this study is the absence of exact reproducibility of the TDC benchmarks even for the perfectly described models. We believe that it originates from the absence of explicit data set versioning within the TDC benchmark suite. The platform does not provide fixed data set releases, version identifiers, check sums or detailed changelogs describing updates to data composition and/or processing, so the data set snapshot used to generate leaderboard metrics may differ from the version available at the time of reproduction. Consequently, some discrepancies between reproduced and leaderboard results may reflect data set evolution rather than genuine methodological advantages or limitations of the models. More broadly, the lack of transparent versioning also reduces the practical usability of the benchmark, as it complicates cross-study comparisons and makes it difficult to determine whether performance discrepancies arise from the modeling pipeline features or from undocumented changes in the underlying data.

A complementary, data set-level limitation of TDC has been documented by several independent studies. Bento et al.^48^ showed that without a structure-standardization step (salt and solvent stripping, charge neutralization, tautomer canonicalization, stereochemistry sanitization), simple canonical-SMILES matching systematically underestimates the number of duplicate molecules in chemical data sets − exactly the failure mode we observed for MiniMol’s filtering of its pretraining corpus against the TDC test sets (Figure 1d). Building on the same principle, Kamuntavičius et al.^37^ applied a structured deduplication protocol to several TDC ADMET end points and reported that a nontrivial fraction of compounds within the official splits are duplicated and, in a number of cases, carry conflicting labels. Gadaleta et al.^33^ reached analogous conclusions over a broader set of TK/PC data sets, where curated and deduplicated versions led to noticeably different model rankings. Together, these studies indicate that residual structural and label-level inconsistencies inside the official TDC splits can inflate test-set metrics for *all* benchmarked models − independent of any specific architecture or training pipeline. We did not recurate the TDC splits ourselves, in order to keep our reevaluation directly comparable with the published leaderboard values. However, we view explicit, versioned curation of the underlying data sets as one of the most important prerequisites for the next generation of ADMET benchmarks, alongside the methodological recommendations discussed below. A systematic structure-standardization pass over the official TDC splits (salt and solvent stripping, charge neutralization, tautomer canonicalization, and stereochemistry sanitization) is the natural way to quantify how much residual duplication remains inside the benchmark itself, and we regard it as a distinct and worthwhile companion study to the model-level audit reported here.

A broader picture of how data set-level shortcomings limit benchmark reliability has been laid out by Walters,^49^ who argues that widely used public ML benchmarks, including TDC, share a recurring set of problems that go beyond duplicate compounds. These include invalid or ambiguously specified chemical structures (e.g., uncharged tetravalent nitrogens that cheminformatics toolkits cannot parse), the absence of a uniform protonation/salt-handling standard, undefined or racemic stereocenters in compounds where stereochemistry strongly affects activity, heterogeneous measurements aggregated across laboratories and assay protocols (a particular concern for IC50-derived end points), unrealistically wide dynamic ranges and arbitrary classification thresholds, residual label-conflicting duplicates, contamination of “active” sets by assay-artifact-prone chemotypes, and the use of clinically or biologically heterogeneous end points whose measurement variability may itself exceed the predictive signal. The likely consequence, emphasized in the same essay, is that part of what looks like model performance on such benchmarks reflects data set idiosyncrasies rather than genuine generalization. A systematic Walters-style audit of all 22 TDC ADMET end points (examining invalid or ambiguous structures, protonation and salt handling, undefined stereochemistry, heterogeneous assay provenance, and label-conflicting duplicates end point by end point) lies outside the end-user scope of the present work, but we consider it one of the most valuable directions for follow-up and hope the audit reported here helps motivate it.

TDC occupies a distinctive leading position among publicly available resources for ADMET prediction benchmarking, while other platforms implement somewhat different benchmarking models. DREAM^52^ and SAMPL^53^ rely primarily on time-limited blind challenges with centralized evaluation on hidden data; CodaBench^54^ offers infrastructure for containerized benchmarks but does not maintain its own ADMET leaderboard; MoleculeNet^55^ standardizes data sets and metrics but does not run a continuous ranking; PharmaBench^56^ emphasizes reproducible workflows but lacks a community-wide public leaderboard; OpenADMET^57^ maintains an open repository of ADMET models while developing supporting tools for training and model-to-model comparison. Therefore, none of these alternatives combines broad ADMET coverage with a continuously maintained public leaderboard in the way TDC does.

At the same time TDC leaderboards also demonstrate significant shortcomings, which have to be addressed in the next generation of ADMET benchmarks. The test set should not be publicly available to avoid deliberate or accidental model fitting toward it. The data set should be strictly versioned with a distinct checksum assigned to each release or snapshot. Reported model results should be tied to this checksum to allow exact reproducibility. The model authors should submit the models rather than their results, complete with the inference environments in a standardized format. This will allow automatic quality control, reproducibility and proper versioning of the leaderboard.

Our results indicate that TDC leaderboards are useful as a reference point, but high positions should not be interpreted as direct evidence of a model’s predictive strength. Under conditions of fully open test data, TDC leaderboard results can be highly sensitive to various forms of data leakage, including apparent or hidden optimization of models for a specific benchmark sample.

## Supplementary Material



## Data Availability

The code and data used for benchmarking our in-house models and selected TDC leaderboard models is available in the GitHub repository https://github.com/receptor-ai/tdc-admet-bench. The snapshot of the TDC data used for benchmarking is available at https://zenodo.org/records/20180944.

## Notes

Table S1: main characteristics of the 22 TDC ADMET end points; Table S2: molecular fingerprint types evaluated as candidates for sequential forward selection; Table S3: metrics and ranks of the in-house models across the TDC ADMET end points; Table S4: metrics and ranks of the deliberately overfitted in-house models across the TDC ADMET end points; environment-setup and runtime error logs for the ADMETrix, SimGCN, and ZairaChem models (PDF)
