# Supplementary material for: An End-User Audit of Reproducibility, Data Leakage, and Overfitting of the Top-Ranked ADMET Prediction Models in TDC Leaderboards
Source: J Chem Inf Model. 2026 Jul 2;66(14):8045–58. doi: 10.1021/acs.jcim.6c00819 (PMC13417885; doi:10.1021/acs.jcim.6c00819)
Supplement: Supplementary file 1 [file ci6c00819_si_001.pdf]

An End-User Audit of Reproducibility, Data Leakage, and Overfitting of the Top-Ranked ADMET Prediction Models in TDC Leaderboards

Supplementary Materials

Ihor Koleiev [1,2], Roman Stratiichuk [1,3], Nazar Shevchuk [1], Mykola Melnychenko [1], Alex Nyporko [7], Daniil Todoryshyn [1], Vladyslav Husak [1,4], Sergii Starosyla [1], Semen Yesylevskyy [1,2,5,6,], Alan Nafiiev [1].*

[1] Receptor.AI Inc., 20-22 Wenlock Road, London N1 7GU, United Kingdom.

[2] Department of Physics of Biological Systems, Institute of Physics of The National Academy of Sciences of Ukraine, 46 Nauky Ave., 03038, Kyiv, Ukraine.

[3] Department of Biophysics and Medical Informatics, Educational and Scientific Centre "Institute of Biology and Medicine", Taras Shevchenko Kyiv National University, 64 Volodymyrska Str., 01601, Kyiv, Ukraine.

[4] Department of Cellular, Computational and Integrative Biology, The University of Trento, Via Sommarive 9, 38123 Povo (Trento), Italy.

[5] Institute of Organic Chemistry and Biochemistry, Czech Academy of Sciences, CZ-166 10 Prague 6, Czech Republic.

[6] Department of Physical Chemistry, Faculty of Science, Palacký University Olomouc, 17. listopadu 12, 771 46 Olomouc, Czech Republic.

[7] Taras Shevchenko Kyiv National University, 64 Volodymyrska Str., 01601, Kyiv, Ukraine.

* Email: semen.yesylevskyy@uochb.cas.cz

Table S1. Main characteristics of TDC ADMET endpoints

Category	Benchmark	Task	Metric	Train Size	Test Size	Description
Absorption	caco2_wang	Regression	MAE	706	176	Caco-2 cell effective permeability
	bioavailability_ma	Classification	AUROC	475	119	Oral bioavailability (F > 20%)
	lipophilicity_astrazeneca	Regression	MAE	3,360	840	Lipophilicity (log D7.4)
	solubility_aqsoldb	Regression	MAE	7,831	1,958	Aqueous solubility
Distribution	hia_hou	Classification	AUROC	475	119	Human intestinal absorption
	pgp_broccatelli	Classification	AUROC	1,028	257	P-glycoprotein inhibition
	bbb_martins	Classification	AUROC	1,560	390	Blood-brain barrier penetration
	ppbr_az	Regression	MAE	1,520	380	Plasma protein binding rate
Metabolism	vdss_lombardo	Regression	Spearman	968	242	Volume of distribution
	cyp2c9_veith	Classification	AUPRC	10,192	2,548	CYP2C9 inhibition
	cyp2d6_veith	Classification	AUPRC	11,127	2,782	CYP2D6 inhibition
	cyp3a4_veith	Classification	AUPRC	10,164	2,541	CYP3A4 inhibition
	cyp2c9_substrate_carbonmangels	Classification	AUPRC	525	131	CYP2C9 substrate
	cyp2d6_substrate_carbonmangels	Classification	AUPRC	571	143	CYP2D6 substrate
	cyp3a4_substrate_carbonmangels	Classification	AUROC	534	134	CYP3A4 substrate
Excretion	half_life_obach	Regression	Spearman	534	134	Terminal half-life
	clearance_hepatocyte_az	Regression	Spearman	870	218	Hepatocyte intrinsic clearance
	clearance_microsome_az	Regression	Spearman	1,020	255	Microsome intrinsic clearance
Toxicity	ld50_zhu	Regression	MAE	5,931	1,483	Acute oral toxicity LD50
	herg	Classification	AUROC	538	135	hERG cardiotoxicity
	ames	Classification	AUROC	6,265	1,566	AMES mutagenicity
	dili	Classification	AUROC	382	96	Drug-induced liver injury

Table S2. Fingerprint types - candidates for sequential forward selection

Category	Fingerprint	Abbreviation	Size	Parameters
**Binary (folded)**	Extended Connectivity Fingerprint	ecfp	1024	radius=2
	Functional Connectivity Fingerprint	fcfp	1024	radius=2
	Avalon Fingerprint	avalon	1024	-
	RDKit Fingerprint	rdkit	1024	-
	Topological Torsion Fingerprint	topological	1024	-
	Atom Pair Fingerprint	atompair	1024	-
	Pattern Fingerprint	pattern	1024	-
	Layered Fingerprint	layered	1024	-
	SMILES Extended Connectivity Fingerprint	secfp	1024	-
**Count-based (folded)**	ECFP with counts	ecfp-count	1024	radius=2
	FCFP with counts	fcfp-count	1024	radius=2
	RDKit with counts	rdkit-count	1024	-
	Topological Torsion with counts	topological-count	1024	-
	Atom Pair with counts	atompair-count	1024	-
**Fixed-size**	MACCS Keys	maccs	167	-
	Extended Reduced Graph	erg	315	-
	E-State Fingerprint	estate	79	-
	RDKit 2D Descriptors (vectorized)	desc2D	223	-
	Mordred 2D Descriptors (vectorized)	mordred	1613	ignore_3D=True
	CATS2D	cats2D	189	-
	Scaffold Keys	scaffoldkeys	42	-
	SKeys	skeys	42	-

Errors when setting up the required execution environment for the ADMETRix model.

```
Pip subprocess error:

ERROR: Ignored the following versions that require a different python
version: 0.12.0 Requires-Python >=3.11; 0.12.0rc1 Requires-Python >=3.11;
0.12.0rc2 Requires-Python >=3.11; 0.12.0rc3 Requires-Python >=3.11;
0.12.0rc4 Requires-Python >=3.11; 0.12.1 Requires-Python >=3.11; 0.12.2
Requires-Python >=3.11; 0.12.3 Requires-Python >=3.11; 0.12.4 Requires-
Python >=3.11; 0.12.5 Requires-Python >=3.11; 0.12.6 Requires-
Python >=3.11; 0.12.7 Requires-Python >=3.11; 0.36.0 Requires-
Python >=3.6,<3.10; 0.37.0 Requires-Python >=3.7,<3.10; 0.52.0 Requires-
Python >=3.6,<3.9; 0.52.0rc3 Requires-Python >=3.6,<3.9; 0.53.0 Requires-
Python >=3.6,<3.10; 0.53.0rc1.post1 Requires-Python >=3.6,<3.10; 0.53.0rc2
Requires-Python >=3.6,<3.10; 0.53.0rc3 Requires-Python >=3.6,<3.10; 0.53.1
Requires-Python >=3.6,<3.10; 0.54.0 Requires-Python >=3.7,<3.10; 0.54.0rc2
Requires-Python >=3.7,<3.10; 0.54.0rc3 Requires-Python >=3.7,<3.10; 0.54.1
Requires-Python >=3.7,<3.10; 0.7 Requires-Python >=3.6,<3.7; 0.8 Requires-
Python >=3.6,<3.7; 1.3.3 Requires-Python >=3.11; 1.6.1.dev0 Requires-
Python >=3.7,<3.9; 1.7.0 Requires-Python >=3.7,<3.9; 1.7.1 Requires-
Python >=3.7,<3.9; 2.0.0 Requires-Python >=3.11; 2.0.0rc1 Requires-
Python >=3.11; 2.0.1 Requires-Python >=3.11; 2.0.2 Requires-Python >=3.11;
2.0.3 Requires-Python >=3.11; 2.0.4 Requires-Python >=3.11; 2.0.5 Requires-
Python >=3.11; 2.1.0 Requires-Python >=3.11; 2.1.1 Requires-Python >=3.11;
2.1.2 Requires-Python >=3.11; 2.2.0 Requires-Python >=3.11; 2.2.1 Requires-
Python >=3.11; 2.3.0 Requires-Python >=3.11; 2.3.1 Requires-Python >=3.11;
2.3.2 Requires-Python >=3.11; 2.3.3 Requires-Python >=3.11; 2.3.4 Requires-
Python >=3.11; 2.3.5 Requires-Python >=3.11; 2.4.0 Requires-Python >=3.11;
2.4.0rc1 Requires-Python >=3.11; 2.4.1 Requires-Python >=3.11; 3.0.0rc0
Requires-Python >=3.11; 3.0.0rc1 Requires-Python >=3.11; 3.5 Requires-
Python >=3.11; 3.5rc0 Requires-Python >=3.11; 3.6 Requires-Python >=3.11;
3.6.1 Requires-Python >=3.11,!=3.14.1; 3.6rc0 Requires-Python >=3.11; 9.0.0
Requires-Python >=3.11; 9.0.0b1 Requires-Python >=3.11; 9.0.0b2 Requires-
Python >=3.11; 9.0.1 Requires-Python >=3.11; 9.0.2 Requires-Python >=3.11;
9.1.0 Requires-Python >=3.11; 9.2.0 Requires-Python >=3.11; 9.3.0 Requires-
Python >=3.11; 9.4.0 Requires-Python >=3.11; 9.5.0 Requires-Python >=3.11;
9.6.0 Requires-Python >=3.11; 9.7.0 Requires-Python >=3.11; 9.8.0 Requires-
Python >=3.11; 9.9.0 Requires-Python >=3.11

ERROR: Could not find a version that satisfies the requirement python-
graphviz==0.20.3 (from versions: none)

ERROR: No matching distribution found for python-graphviz==0.20.3

failed
```

Errors when setting up the required execution environment for the SimGCN model.

```
Traceback (most recent call last):
 File "/home/nazar/Desktop/TDC/SimGCN-TDC/run.py", line 5, in <module>
   from katanaHLS.data.dataset import SIPGraph
 File "/home/nazar/Desktop/TDC/SimGCN-TDC/katanaHLS/data/dataset.py", line
8, in <module>
   from torch_geometric.data import InMemoryDataset, Data
File "/home/nazar/.pyenv/versions/3.12.3/lib/python3.12/site-
packages/torch_geometric/__init__.py", line 7, in <module>
   import torch_geometric.data
```

```
File "/home/nazar/.pyenv/versions/3.12.3/lib/python3.12/site-packages/torch_geometric/data/__init__.py", line 1, in <module>
   from .data import Data
 File "/home/nazar/.pyenv/versions/3.12.3/lib/python3.12/site-packages/torch_geometric/data/data.py", line 3, in <module>
   from torch_geometric.typing import OptTensor, NodeType, EdgeType
 File "/home/nazar/.pyenv/versions/3.12.3/lib/python3.12/site-packages/torch_geometric/typing.py", line 4, in <module>
   from torch_sparse import SparseTensor
ModuleNotFoundError: No module named 'torch_sparse'
 Cant install torch sparse
Collecting torch-sparse
  Using cached torch_sparse-0.6.18.tar.gz (209 kB)
  Installing build dependencies ... done
  Getting requirements to build wheel ... error
  error: subprocess-exited-with-error
 × Getting requirements to build wheel did not run successfully.
  │ exit code: 1
  ╰─> [20 lines of output]
      Traceback (most recent call last):
        File "/home/nazar/.pyenv/versions/3.12.3/lib/python3.12/site-packages/pip/_vendor/pyproject_hooks/_in_process/_in_process.py", line 389, in <module>
          main()
        File "/home/nazar/.pyenv/versions/3.12.3/lib/python3.12/site-packages/pip/_vendor/pyproject_hooks/_in_process/_in_process.py", line 373, in main
          json_out["return_val"] = hook(**hook_input["kwargs"])
                                   ^^^^^^^^^^^^^^^^^^^^^^^^^^^^
        File "/home/nazar/.pyenv/versions/3.12.3/lib/python3.12/site-packages/pip/_vendor/pyproject_hooks/_in_process/_in_process.py", line 143, in get_requires_for_build_wheel
          return hook(config_settings)
                 ^^^^^^^^^^^^^^^^^^^^^
        File "/tmp/pip-build-env-1ooew3s2/overlay/lib/python3.12/site-packages/setuptools/build_meta.py", line 331, in get_requires_for_build_wheel
          return self._get_build_requires(config_settings, requirements=[])

^^^^^^^^^^^^^^^^^^^^^^^^^^^^^^^^^^^^^^^^^^^^^^^^^^^^^^^^^^
        File "/tmp/pip-build-env-1ooew3s2/overlay/lib/python3.12/site-packages/setuptools/build_meta.py", line 301, in _get_build_requires
          self.run_setup()
        File "/tmp/pip-build-env-1ooew3s2/overlay/lib/python3.12/site-packages/setuptools/build_meta.py", line 512, in run_setup
          super().run_setup(setup_script=setup_script)
        File "/tmp/pip-build-env-1ooew3s2/overlay/lib/python3.12/site-packages/setuptools/build_meta.py", line 317, in run_setup
          exec(code, locals())
        File "<string>", line 8, in <module>
      ModuleNotFoundError: No module named 'torch'
      [end of output]
 note: This error originates from a subprocess, and is likely not a problem with pip.
ERROR: Failed to build 'torch-sparse' when getting requirements to build wheel
```

In /usr/lib/x86_64-linux-gnu run something like sudo ln -s libtiff.so.6 libtiff.so.5
doesn't work

ZairaChem runtime errors

```
(base) nazar_shevchuk_receptor_ai@admet-predict:~/zaira-chem$ conda
activate zairachem
(zairachem) nazar_shevchuk_receptor_ai@admet-predict:~/zaira-
chem$ zairachem --help
2026-02-06 02:24:30.101954: I
tensorflow/core/platform/cpu_feature_guard.cc:193] This TensorFlow binary
is optimized with oneAPI Deep Neural Network Library (oneDNN) to use the
following CPU instructions in performance-critical operations:  AVX2 FMA
To enable them in other operations, rebuild TensorFlow with the
appropriate compiler flags.
2026-02-06 02:24:30.314378: E
tensorflow/stream_executor/cuda/cuda_blas.cc:2981] Unable to register
cuBLAS factory: Attempting to register factory for plugin cuBLAS when one
has already been registered
2026-02-06 02:24:31.237772: W
tensorflow/stream_executor/platform/default/dso_loader.cc:64] Could not
load dynamic library 'libnvinfer.so.7'; dlerror: libnvinfer.so.7: cannot
open shared object file: No such file or directory
2026-02-06 02:24:31.237930: W
tensorflow/stream_executor/platform/default/dso_loader.cc:64] Could not
load dynamic library 'libnvinfer_plugin.so.7'; dlerror:
libnvinfer_plugin.so.7: cannot open shared object file: No such file or
directory
2026-02-06 02:24:31.237952: W
tensorflow/compiler/tf2tensorrt/utils/py_utils.cc:38] TF-TRT Warning:
Cannot dlopen some TensorRT libraries. If you would like to use Nvidia GPU
with TensorRT, please make sure the missing libraries mentioned above are
installed properly.
Traceback (most recent call last):
  File
"/home/nazar_shevchuk_receptor_ai/miniconda3/envs/zairachem/bin/zairachem"
, line 3, in <module>
    from zairachem.cli import cli
  File "/home/nazar_shevchuk_receptor_ai/zaira-
chem/zairachem/cli/__init__.py", line 1, in <module>
    from .create_cli import create_cli
  File "/home/nazar_shevchuk_receptor_ai/zaira-
chem/zairachem/cli/create_cli.py", line 1, in <module>
    from .cmd import Command
  File "/home/nazar_shevchuk_receptor_ai/zaira-chem/zairachem/cli/cmd.py",
line 4, in <module>
    from .commands.estimate import estimate_cmd
  File "/home/nazar_shevchuk_receptor_ai/zaira-
chem/zairachem/cli/commands/estimate.py", line 7, in <module>
    from ...estimators.pipe import EstimatorPipeline
  File "/home/nazar_shevchuk_receptor_ai/zaira-
chem/zairachem/estimators/pipe.py", line 10, in <module>
    from .from_individual_full_descriptors_tabpfn.pipe import (
  File "/home/nazar_shevchuk_receptor_ai/zaira-
chem/zairachem/estimators/from_individual_full_descriptors_tabpfn/pipe.py"
, line 1, in <module>
    from .estimate import Estimator
  File "/home/nazar_shevchuk_receptor_ai/zaira-
chem/zairachem/estimators/from_individual_full_descriptors_tabpfn/estimate
.py", line 12, in <module>
    from ...automl.binarytabpfn import TabPFNBinaryClassifier
  File "/home/nazar_shevchuk_receptor_ai/zaira-
chem/zairachem/automl/binarytabpfn.py", line 5, in <module>
```

```
    from tabpfn import TabPFNClassifier
  File
"/home/nazar_shevchuk_receptor_ai/miniconda3/envs/zairachem/lib/python3.10
/site-packages/tabpfn/__init__.py", line 1, in <module>
    from tabpfn.scripts.transformer_prediction_interface import
TabPFNClassifier
  File
"/home/nazar_shevchuk_receptor_ai/miniconda3/envs/zairachem/lib/python3.10
/site-packages/tabpfn/scripts/transformer_prediction_interface.py", line
19, in <module>
    from tabpfn.scripts.model_builder import load_model,
load_model_only_inference
  File
"/home/nazar_shevchuk_receptor_ai/miniconda3/envs/zairachem/lib/python3.10
/site-packages/tabpfn/scripts/model_builder.py", line 4, in <module>
    from tabpfn.transformer import TransformerModel
  File
"/home/nazar_shevchuk_receptor_ai/miniconda3/envs/zairachem/lib/python3.10
/site-packages/tabpfn/transformer.py", line 9, in <module>
    from tabpfn.layer import TransformerEncoderLayer, _get_activation_fn
  File
"/home/nazar_shevchuk_receptor_ai/miniconda3/envs/zairachem/lib/python3.10
/site-packages/tabpfn/layer.py", line 5, in <module>
    from torch.nn.modules.transformer import _get_activation_fn, Module,
Tensor, Optional, MultiheadAttention, Linear, Dropout, LayerNorm
ImportError: cannot import name 'Optional' from
'torch.nn.modules.transformer'
(/home/nazar_shevchuk_receptor_ai/miniconda3/envs/zairachem/lib/python3.10
/site-packages/torch/nn/modules/transformer.py)
```

Table S3. Metrics and ranks of in-house models on different TDC ADMET endpoints

benchmark	metric name	baseline metric	baseline rank	sfs metric	sfs rank	hpo metric	hpo rank	total	features	best_params
caco2_wang	MAE	0.3067	9	0.2889	7	0.2875	7	24	mol2vec, filtered_descs, topological-count, maccs	n_estimators 200 learning_rate 0.1 max_depth -1 num_leaves 63
bioavailability_ma	AUROC	0.6784	21	0.6744	21	0.6744	21	20	mol2vec, topological-count, rdkit-count, topological, layered	n_estimators 100 learning_rate 0.1 max_depth -1 num_leaves 31
lipophilicity_astrazeneca	MAE	0.5701	13	0.5225	7	0.5108	5	21	mol2vec, filtered_descs, avalon-count, ecfp-count, estate	n_estimators 800 learning_rate 0.05 max_depth 5 num_leaves 15
solubility_aqsoldb	MAE	0.813	7	0.7932	7	0.7808	5	18	mol2vec, mordred, avalon-count, desc2D, topological-count	n_estimators 400 learning_rate 0.03 max_depth -1 num_leaves 63
hia_hou	AUROC	0.9901	2	0.9938	1	0.9938	1	20	mol2vec, mordred, ecfp-count, maccs, pattern	
pgp_broccatelli	AUROC	0.9123	8	0.8968	10	0.902	10	16	mol2vec, filtered_descs, erg, atompair-count, estate	n_estimators 800 learning_rate 0.1

										max_depth 9 num_leaves 127
bbb_martins	AUROC	0.9199	8	0.9225	2	0.8884	17	26	mol2vec, erg, pattern	n_estimators 100 learning_rate 0.01 max_depth 9 num_leaves 31
ppbr_az	MAE	8.4496	9	8.4795	10	7.9457	7	20	mol2vec, mordred, pattern, desc2D	n_estimators 400 learning_rate 0.05 max_depth 5 num_leaves 15
vdss_lombardo	Spearman	0.3356	17	0.3368	17	0.4949	11	19	mol2vec, desc2D, rdkit-count, topological, mordred	n_estimators 100 learning_rate 0.01 max_depth -1 num_leaves 63
cyp2c9_veith	AUPRC	0.7613	9	0.7883	6	0.7922	5	20	mol2vec, mordred, rdkit-count, avalon-count, scaffoldkeys	n_estimators 800 learning_rate 0.1 max_depth 9 num_leaves 63
cyp2d6_veith	AUPRC	0.7002	7	0.7069	7	0.7178	7	19	mol2vec, filtered_descs, ecfp-count, topological, avalon-count	n_estimators 800 learning_rate 0.05 max_depth -1 num_leaves 63
cyp3a4_veith	AUPRC	0.8511	11	0.8683	9	0.8748	9	19	mol2vec, avalon-count, rdkit, cats2D, scaffoldkeys	n_estimators 800 learning_rate 0.05 max_depth 3 num_leaves 15
cyp2c9_substrate_carbonmangels	AUPRC	0.3363	20	0.3459	19	0.3459	19	20	mol2vec, filtered_descs	n_estimators 100 learning_rate 0.1

										max_depth -1 num_leaves 31
cyp2d6_substrate_carbonmangels	AUPRC	0.657	12	0.6842	10	0.6842	10	18	mol2vec, rdkit-count, maccs, desc2D, ecfp	n_estimators 100 learning_rate 0.1 max_depth -1 num_leaves 31
cyp3a4_substrate_carbonmangels	AUROC	0.607	14	0.6384	9	0.6032	15	19	mol2vec, mordred, pattern	n_estimators 200 learning_rate 0.01 max_depth 3 num_leaves 31
half_life_obach	Spearman	0.3173	11	0.2718	12	0.3927	9	20	mol2vec, rdkit-count, avalon, atompair-count	n_estimators 200 learning_rate 0.01 max_depth -1 num_leaves 127
clearance_hepatocyte_az	Spearman	0.3259	16	0.3373	16	0.3353	16	18	mol2vec, filtered_descs, scaffoldkeys, topological-count	n_estimators 100 learning_rate 0.05 max_depth -1 num_leaves 31
clearance_microsome_az	Spearman	0.5432	14	0.5542	14	0.5512	15	20	mol2vec, filtered_descs, rdkit-count, layered, topological	n_estimators 400 learning_rate 0.1 max_depth -1 num_leaves 31
ld50_zhu	MAE	0.6212	6	0.61	6	0.5866	4	22	mol2vec, mordred, avalon, rdkit, atompair-count	n_estimators 400 learning_rate 0.05 max_depth 9 num_leaves 31

herg	AUROC	0.815	11	0.8032	11	0.8032	11	20	mol2vec, filtered_descs, maccs, erg, cats2D	n_estimators 100 learning_rate 0.1 max_depth -1 num_leaves 31
ames	AUROC	0.8255	13	0.8422	10	0.7942	17	20	mol2vec, pattern, layered, cats2D, rdkit	n_estimators 100 learning_rate 0.01 max_depth -1 num_leaves 15
dili	AUROC	0.8787	13	0.86	16	0.86	16	20	mol2vec, avalon, atompair, pattern	n_estimators 100 learning_rate 0.1 max_depth -1 num_leaves 31

Table S4. Metrics and ranks of deliberately overfitted in-house models on different TDC ADMET endpoints

**benchmark**	**metric name**	**baseline metric**	**baseline rank**	**sfs metric**	**sfs rank**	**hpo metric**	**hpo rank**	**total**	**features**	**best_params**
caco2_wang	MAE	0.3067	9	0.2694	2	0.2694	2	24	mol2vec, desc2D, erg, filtered_descs	n_estimators 100 learning_rate 0.1
bioavailability_ma	AUROC	0.6784	21	0.7147	21	0.7147	21	20	mol2vec, topological, atompair, rdkit-count	n_estimators 100 learning_rate 0.1
lipophilicity_astrazeneca	MAE	0.5701	13	0.5345	8	0.5112	5	21	mol2vec, filtered_descs, avalon-count, layered, atompair-count	n_estimators 300 learning_rate 0.1

solubility_aqsoldb	MAE	0.813	7	0.7883	5	0.7735	3	18	mol2vec, filtered_descs, mordred	n_estimators 300<br>learning_rate 0.1
hia_hou	AUROC	0.9901	2	0.9951	1	0.9951	1	20	mol2vec, erg, maccs, pattern, mordred	
pgp_broccatelli	AUROC	0.9123	8	0.9188	7	0.9202	7	16	mol2vec, mordred, rdkit-count, cats2D	n_estimators 200<br>learning_rate 0.2
bbb_martins	AUROC	0.9199	8	0.9376	1	0.9376	1	26	mol2vec, atompair-count, cats2D, atompair	
ppbr_az	MAE	8.4496	9	7.779	5	7.9893	7	20	mol2vec, filtered_descs, rdkit	n_estimators 300<br>learning_rate 0.05
vdss_lombardo	Spearman	0.3356	17	0.4909	13	0.4664	15	19	mol2vec, avalon-count, filtered_descs, desc2D	n_estimators 100,<br>learning_rate 0.1
cyp2c9_veith	AUPRC	0.7613	9	0.792	5	0.792	5	20	mol2vec, mordred, avalon-count, layered, secfp	n_estimators 100<br>learning_rate 0.1
cyp2d6_veith	AUPRC	0.7002	7	0.7197	5	0.7085	7	19	mol2vec, filtered_descs, atompair-count	n_estimators 200<br>learning_rate 0.05
cyp3a4_veith	AUPRC	0.8511	11	0.8733	9	0.8826	5	19	mol2vec, filtered_descs, ecfp-count, layered, pattern	n_estimators 300<br>learning_rate 0.2
cyp2c9_substrate_carbonmangels	AUPRC	0.3363	20	0.3566	18	0.3566	18	20	mol2vec, mordred	n_estimators 100<br>learning_rate 0.1
cyp2d6_substrate_carbonmangels	AUPRC	0.657	12	0.7566	1	0.7566	1	18	mol2vec, desc2D, secfp, avalon-count	

cyp3a4_substrate_carbonmangels	AUROC	0.607	14	0.6658	2	0.6658	2	19	mol2vec, maccs, scaffoldkeys	n_estimators 100<br>learning_rate 0.1
half_life_obach	Spearman	0.3173	11	0.4053	9	0.4089	9	20	mol2vec, mordred, topological-count, rdkit	n_estimators 100<br>learning_rate 0.05
clearance_hepatocyte_az	Spearman	0.3259	16	0.4413	6	0.4413	6	18	mol2vec, secfp, ecfp, atompair	n_estimators 100<br>learning_rate 0.1
clearance_microsome_az	Spearman	0.5432	14	0.6314	1	0.6314	1	20	mol2vec, avalon-count, desc2D, atompair	
ld50_zhu	MAE	0.6212	6	0.5964	5	0.5766	3	22	mol2vec, mordred, atompair-count, avalon, topological-count	n_estimators 300<br>learning_rate 0.1
herg	AUROC	0.815	11	0.8495	6	0.8495	6	20	mol2vec, desc2D, rdkit-count, layered, secfp	n_estimators 100<br>learning_rate 0.1
ames	AUROC	0.8255	13	0.8652	5	0.8723	1	20	mol2vec, mordred, rdkit, avalon-count, topological	n_estimators 300<br>learning_rate 0.1
dili	AUROC	0.8787	13	0.9422	2	0.9391	2	20	mol2vec, mordred, filtered_descs, scaffoldkeys, secfp	n_estimators 200<br>learning_rate 0.05
